# Case report: Cortico-ocular interaction networks in NBA2K

**DOI:** 10.3389/fnetp.2023.1151832

**Published:** 2023-04-11

**Authors:** Andreas Stamatis, Sergi Garcia-Retortillo, Grant B. Morgan, Ana Sanchez-Moreno

**Affiliations:** ^1^ Exercise and Nutrition Sciences, State University of New York, Plattsburgh, NY, United States; ^2^ Health and Exercise Science, Wake Forest University, Winston-Salem, NC, United States; ^3^ Educational Psychology, Baylor University, Waco, TX, United States; ^4^ Health and Sport, University of Girona, Salt, Spain

**Keywords:** curia, EEG, sport neuroscience, pupillometry, brain waves, eye tracking, esports

## Abstract

The sport industry has never seen growth such as eSports’. Using synchronized monitoring of two biological processes on a 25-year-old gamer, we investigated how his brain (*via* EEG) and eyes (*via* pupil dilation) interacted dynamically over time as an integrated network during NBA2K playing time. After the spectral decomposition of the different Brain and Eye signals into seven frequency bands, we calculated the bivariate equal-time Pearson’s cross-correlation between each pair of EEG/Eye spectral power time series. On average, our results show a reorganization of the cortico-muscular network across three sessions (e.g., new interactions, hemispheric asymmetry). These preliminary findings highlight the potential need for individualized, specific, adaptive, and periodized interventions and encourage the continuation of this line of research for the creation of general theories of networks in eSports gaming. Future studies should recruit larger samples, investigate different games, and explore cross-frequency coordination among other key organ systems.

## 1 Introduction

The growth of eSports has been unprecedented (Newman et al., 2022). Esports are video games played in a structured and competitive manner (Nagorsky and Wiemeyer, 2020). These games are categorized into different genres, such as fighting games, first-person shooters, real-time strategy games, and sports simulations.

NBA2K is a popular, E-rated, “Sports Sim” game developed after the game of basketball and more specifically the NBA (Darvin et al., 2021). NBA2K was developed by Visual Concepts and 2K Games to be played on PlayStation, Xbox, Switch, and PC. The player, just like a head coach, can call basketball plays in real time. Also in real time, thanks to recent advancements in technology, we can collect biofeedback (Martin-Niedecken Anna and Schättin, 2020).

Biofeedback can take several forms, such as in the synchronized monitoring of electroencephalogram (EEG) and eye tracking (e.g., pupil dilation). EEG is a non-invasive method to continuously track electrophysiological activity in the brain across time in the form of frequency bands (Hz), such as delta, theta, alpha, beta, and gamma (Carlstedt and Balconi, 2018). The pupil dilation (mm) is considered an indirect biomarker of task demands (van der Wel and van Steenbergen, 2018). Pupillary oscillations have been used in assessing fatigue and alertness (e.g., McLaren et al., 1992 ). EEG and eye-tracking research are not new in NBA2K research (Potwarka et al., 2022). However, how distinct cortical rhythms in the brain dynamically synchronize their activation with the eyes during NBA2K remains unexplored.

The quality of human function is defined by the *coherence* (i.e., a complex, harmonious, synchronized coordination) of all sub-systems (e.g., from molecules to organs), while each functions differently and autonomously (Ho, 2008). In terms of *physiological coherence*, the coherent system operates under an efficient/optimal pattern of activity compared to an erratic/discordant pattern (Tiller et al., 1996). This system-wide, integrative approach to physiological coherence has been proposed by the new field of Network Physiology (Bashan et al., 2012; Ivanov and Bartsch, 2014; Rizzo et al., 2020; Ivanov 2021; Balagué et al., 2022; Chen et al., 2022).

After recognizing the high level of efficiency of coherent systems/networks and the inability of traditional studies to uncover that network information, Network Physiology has been attempting to identify and quantify dynamic networks of diverse systems by avoiding focusing on individual systems. By probing interactions among various systems, the physiological networks are identified. These networks are robust in structure (e.g., topology, function) but transition (e.g., flexible reorganizations) in response to perturbations (e.g., exercise duration).

Drawing from the field of Network Physiology, the purpose of this exploratory, pilot study was to investigate how different cortical rhythms in the brain (*via* EGG) dynamically interact as a network with the eyes (*via* pupil dilation) during NBA2K playing time.

## 2 Methods

The participant was a 25-year-old, right-handed male, who has been playing the NBA2K basketball games for 5+ years, totaling over 4,000 h. The participant was seated in front of a 24″ monitor at roughly 15” distance from a Tobii 5L Eye Tracker (Tobii AB, Sweden). The monitor was connected to a PlayStation 4 (Sony Interactive Entertainment, San Mateo, CA) console. The video was captured through an Elgato HD60 S+ (Elgato Systems GmbH, Germany) capture card at native 60fps. Using corneal reflection (by illuminating the eye with infrared light and measuring the reflection from the cornea and the pupil), pupil diameter data was collected through Tobii 5L Eye Tracker sampling at 120 hz (For information on the accuracy and precision of Tobii 5L, please, see Housholder et al., 2022). EEG data were collected using a Neurosity Crown (Neurosity Inc., San Francisco, CA) EEG device (8 channels, 256 hz). The game was NBA2K 22 Basketball for the PlayStation 4, and the primary game mode was the Recreational Center, which is a 5-on-5 competitive game mode but is not ranked. The participant played 11 times in 1 day with breaks of 10–60 min in between. The recordings took place on 22 April 2022. To investigate the dynamic interactions over time, we chose three recordings (first, sixth, and eleventh), namely, Sessions 1, 2, and 3. Session 1 started at 14:51 (Pacific Time), Session 2 started at 19:12, and Session 3 started at 23:58. They all lasted about 30 min. Lighting was the same throughout and no stimulants were used.

### 2.1 EEG/EYE data acquisition and signal processing

EEG signals were recorded from four brain locations: P03, P04, CP3, and CP4 *via* the Neurosity Crown. That EEG device was connected *via* Wi-Fi and using the LabStreamingLayer (LSL) functionality provided by Neurosity. The LSL stream was connected to CuriaRecorder (Curia LLC, Dublin, CA), which is a fork of the open-source Labrecorder app provided by LSL. The CuriaRecorder is designed to subscribe to and record LSL streams, keeping timestamping and jitter information. The raw data was stored and preprocessed into native XDF format, after which it was run through basic de-noising (i.e., data >4SD of the dataset is removed). Finally, the raw and preprocessed data was made available and a final processing pass was performed. The recording was cut into 15-s intervals. Calculations were performed on these 15-s intervals as single data points. This technique allows long recordings to show the trends over time while being broken into discrete ‘bins’ that calculate the data independently.

For example, in order to collect data on eye movements, the utilized eye tracking device recorded at a sampling rate of 120 Hz. This resulted in a total of 1800 samples being collected within a 15-s processing window, representing the left pupil size in millimeters. The mean of these 1800 values was calculated and stored as the average for that 15-s window. Over the course of an 8-h data collection period, this design resulted in the storage of approximately 3.5 million values for the left pupil size alone. Given the large number of data points collected, and the potential for device noise and cumulative error over the prolonged data collection period, subsampling of the data was employed to “sparsify” the data and facilitate the identification of long-term trends while minimizing short-term variation in any given sample or bin. The subsampled data was extensively tested against the full sample data for both accuracy and various parameters, ranging from 5 to 60-s “chunks”. It was determined that a 15-s processing window provided the desired benefits in processing without altering the shape of the data or the trends that emerged when compared to full sample recordings.

The Tobii 5L Eye Tracker was connected *via* USB3 to the collection system and was using Curia-written LSL broadcasters that take the Tobii datastream from the API and also push it to LSL as a datastream. Using the same acquisition method (i.e., the CuriaRecorder), the Tobii LSL streams were recorded with accurate timestamps and jitter information. They were also stored in LSL native XDF format. The data were preprocessed for outliers, stored as raw and preprocessed data, and finally cut into the same 15-s subintervals for comparison between time points.

Concerning extraction of outliers, the process for cleaning EEG data involves performing a 4-SD removal pass, followed by a 2 SD removal pass. The first step in the process is to calculate a rolling standard deviation of the data with a 1-s window. A threshold is then calculated by taking the 0.3 quantile of these values and multiplying it by 4, resulting in a threshold of 691 μV in this example. Any values (absolute values) exceeding this threshold, plus or minus 691 μV, are rejected and set to 0 for subsequent calculations. For an example channel with 380,000 samples, 866 samples were rejected for exceeding the threshold. The second pass is a standard z-score and 2 SD removal for each channel once the first pass is complete. For pupil data, an exponentially weighted smoothing algorithm is applied to the pupil and gaze values to compensate for the potential introduction of noise by the 120 Hz eye tracking device. This algorithm uses the pandas *exponentially weighted mean* (EWM) functionality, which is similar to a median filter. The smoothing factor used in this implementation is 0.3 (equivalent to moving the smoothing slider in “Tensorflow” for Python). The relevant code section is provided as Supplementary Material.

Of note, in the present study, we employed two distinct methods to address noise (i.e., any undesired or extraneous signal that is not generated by the brain) and outlier (i.e., data points that deviate significantly from the rest of the data and may indicate errors in the recording or data processing) removal from EEG data. The fundamental difference between the two methods is that the 4-SD removal pass was solely directed towards noise reduction in the raw data, while the combined usage of 4-SD and 2-SD removal passes were primarily employed for removing outliers from preprocessed data.

As stated above, the sampling frequency of the Tobii 5L eye tracker and Neurosity Crown EEG were 120 Hz and 256 Hz, respectively. In order to facilitate a comparison between the two datasets, it was necessary to establish a common sampling frequency. Utilizing the “scipy” resample function within the Python programming language, the eye tracking data, stored in a Pandas dataframe, was upsampled to 256 Hz to align with the EEG data. The decision to upsample the eye tracking data rather than downsample the EEG data was made based on the tradeoffs between frequency resolution and the potential loss of information. Therefore, the sampling frequency for both Neurosity Crown and Tobii 5L Eye Tracker was 256 Hz.

Section 2.2–Section 2.5 summarize the main steps for the spectral decomposition and cross-correlations analysis (for a detailed explanation of the entire procedure, please see Garcia-Retortillo and Ivanov 2022; Garcia-Retortillo et al., 2020).

### 2.2 Spectral decomposition

We first segmented the previously pre-processed signals (see Section 2.1. for details) from each brain location and eye into 2-s time windows with a 1-s overlap across each session. Within each 2-s time window, we extracted the spectral power S(f) from each EEG/EYE signal using the ‘pwelch’ function in Matlab, based on the discrete Fourier transform (DFT) and the Welch’s overlapped segment averaging estimator. For each time window, we obtained a spectral power value in bins of 0.5 Hz (fbin) for the range from near 0 Hz to the Nyquist frequency, fN, of 128 Hz (i.e., the half of the sampling frequency of 256 Hz), which renders the total number of spectral estimates, N, for each window of 2 s N = fN * (1/fbin) = 256; that means that *N* = 256 is the number of spectral power data points for each window of 2 s. To probe specific contributions from different frequency bands f_i_ to the spectral power within each 2-s time window of the EEG/Eye signal, we considered seven frequency bands corresponding to the commonly utilized cortical rhythms: δ (0.5–3.5 Hz), θ (4–7.5 Hz), α (8–11.5 Hz), σ (12–15.5 Hz), β (16–19.5 Hz), γ1 (20–33.5 Hz), and γ2 (34–98.5 Hz) (Rizzo et al., 2020; Rizzo et al., 2022). We calculated the average power $<S\left(f\right)>$ across all frequency bins within each frequency: $<S\left(f\right)>:=\sum_{i=1}^{N}S\left(f_{i}\right)/N$ , where $f_{i}$ are all frequencies considered in each frequency band. Thus, we obtained seven time series of EEG/Eye band power $<S\left(f\right)>$ with 1-s resolution for each Brain Location/Eye.

### 2.3 Cross-correlations between EEG/EYE spectra of different frequency bands

**FIGURE 1:**
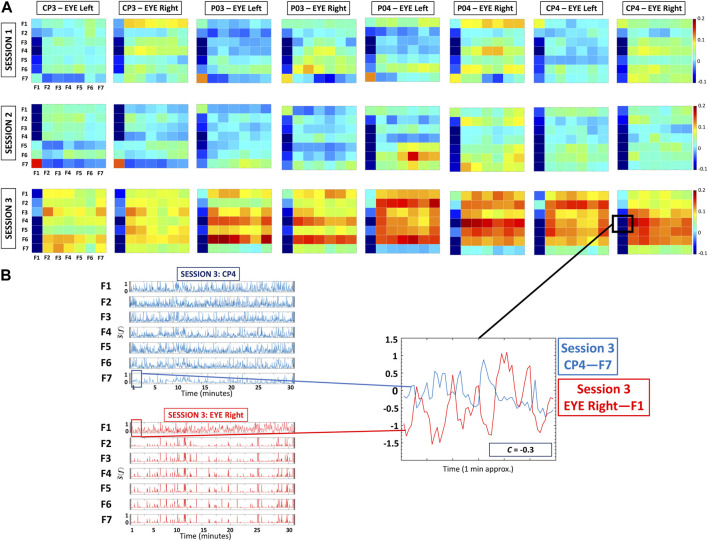
Cortico-ocular cross-correlation matrices during Session 1, Session 2, and Session 3. **(A)** Each matrix represents a given sub-network (i.e., pair of Brain location-Eye): CP3-Eye Left, CP3-Eye Right, P03-Eye Left, P03-Eye Right, P04-Eye Left, P04-Eye Right, CP4-Eye Left, and CP4-Eye Right). Matrix elements represent pairwise coupling strength between the 7 frequency bands of one Brain Location with the same bands derived from one eye. **(B)** Normalized spectral power of frequency bands at CP4 EEG and Eye Right channels (left panels). The right panel shows a 60-s segment for F7 in CP4 and F1 in Eye Right—note that these time series present an anticorrelated behavior as indicated by the corresponding matrix element within the CP4-Eye Right cross-correlation matrix in **(A)**.

Based on the exploratory nature of this research (e.g., investigating the linear relationship between two time series data sets), for each protocol session and each pair of Brain location-Eye (a total of eight different Brain Location/Eye combinations), we calculated the bivariate equal-time Pearson’s cross-correlation for all pairs of time series representing EEG/Eye spectral power in the frequency bands f_i_, where i = 1, … ,7. This led to 49 (7 × 7) cross-correlation values C_i,j_ for each pair of Brain Location-Eye, as shown in the cortico-ocular cross-correlation matrices (Figure 1). The cross-correlation values range from C_i,j_ = −1 (fully anti-correlated) to C_i,j_ = 1 (fully correlated), with C_i,j_ = 0 indicating the absence of a linear relationship between the power spectra of two EEG/EYE frequency bands.

### 2.4 Cortico-ocular cross-correlation matrices

Cortico-ocular cross-correlation matrices represent pairwise coupling strength between the seven frequency bands f_i_ of one brain location with the same bands derived from one Eye (i.e., eight distinct pairs: CP3-Eye Left, CP3-Eye Right, P03-Eye Left, P03-Eye Right, P04-Eye Left, P04-Eye Right, CP4-Eye Left, and CP4-Eye Right) during a given session (Figure 1A). Matrix elements indicate the coupling strength between the band-wise power of EEG signal in a given Brain Location and the pupil diameter dynamics in one Eye.

### 2.5 Cortico-ocular interaction networks

**FIGURE 2:**
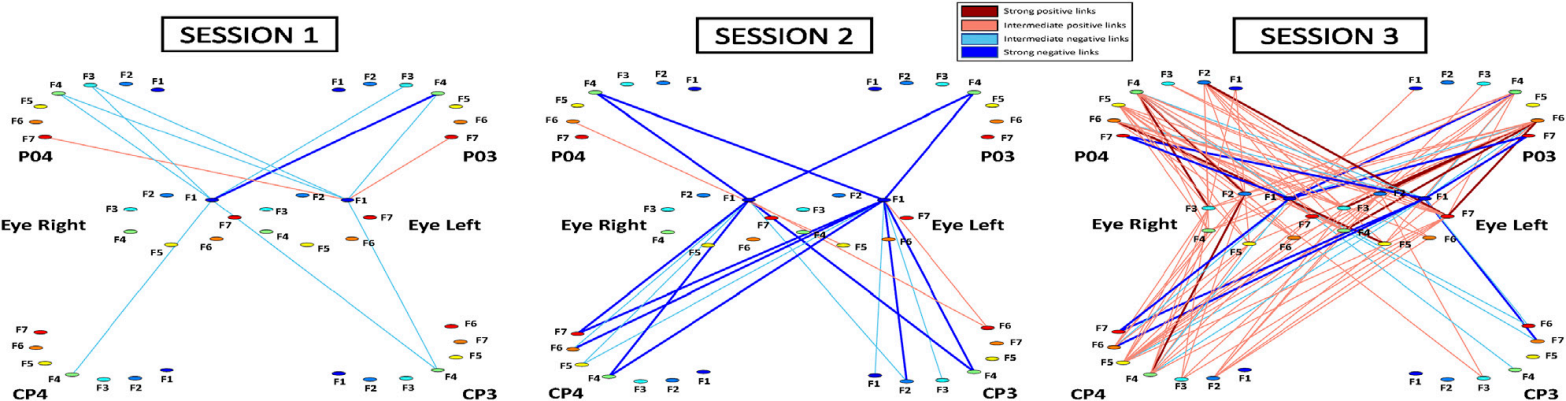
Cortico-ocular interaction networks during Session 1, Session 2, and Session 3. Network maps are obtained based on the cross-correlation matrices shown in Figure 1. Network links correspond to the matrix elements and represent the coupling strength between distinct frequency bands of one brain location with the same bands derived from one eye. Each brain location is shown as a semi-circle and eyes are represented as circles, where color nodes represent frequency bands (Section 2.2, *Methods*). Links strength is marked by line color and width: positive links (C_i,j_ > 0.17; dark red); weak positive links (0.12 < C_i,j_ < 0.17; light red); negative links (C_i,j_ < −0.17; dark blue)) and weak negative links (−0.17 < C_i,j_ < −0.12; light red). Links corresponding to cross-correlation values −0.1< C_ij_ < 0.1 (Figure 2) are not shown in the network maps.

**FIGURE 3:**
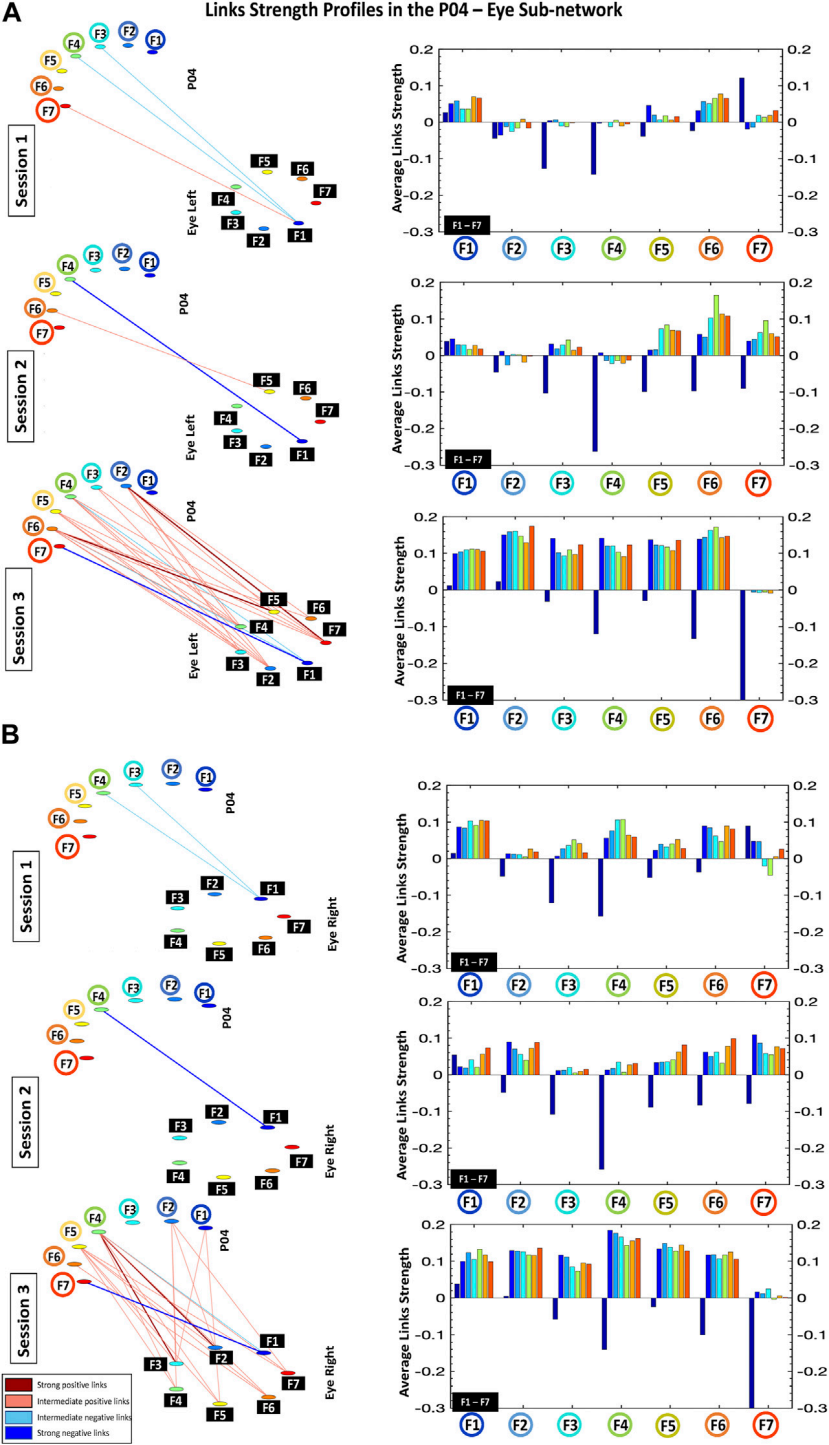
Links strength profiles in the P04-Eye Left **(A)** and P04-Eye Right **(B)** sub-networks. Left panels: dynamic cortico-ocular networks where network links show the coupling strength (degree of synchronous activity) for the P04-Eye sub-networks. Right panels: the P04-Eye sub-networks topology is defined by basic modules, each representing the interaction of a given frequency band from P04 with all frequency bands from Eye Left/Eye Right. Frequency bands of P04 are marked by circles on the horizontal axis of each bar chart, and the frequency bands of the Eye Left/Eye Right are marked by black squares within each module. Bars color in each profile corresponds to the color of the node associated with a given frequency band in the P04 brain location.

**FIGURE 4:**
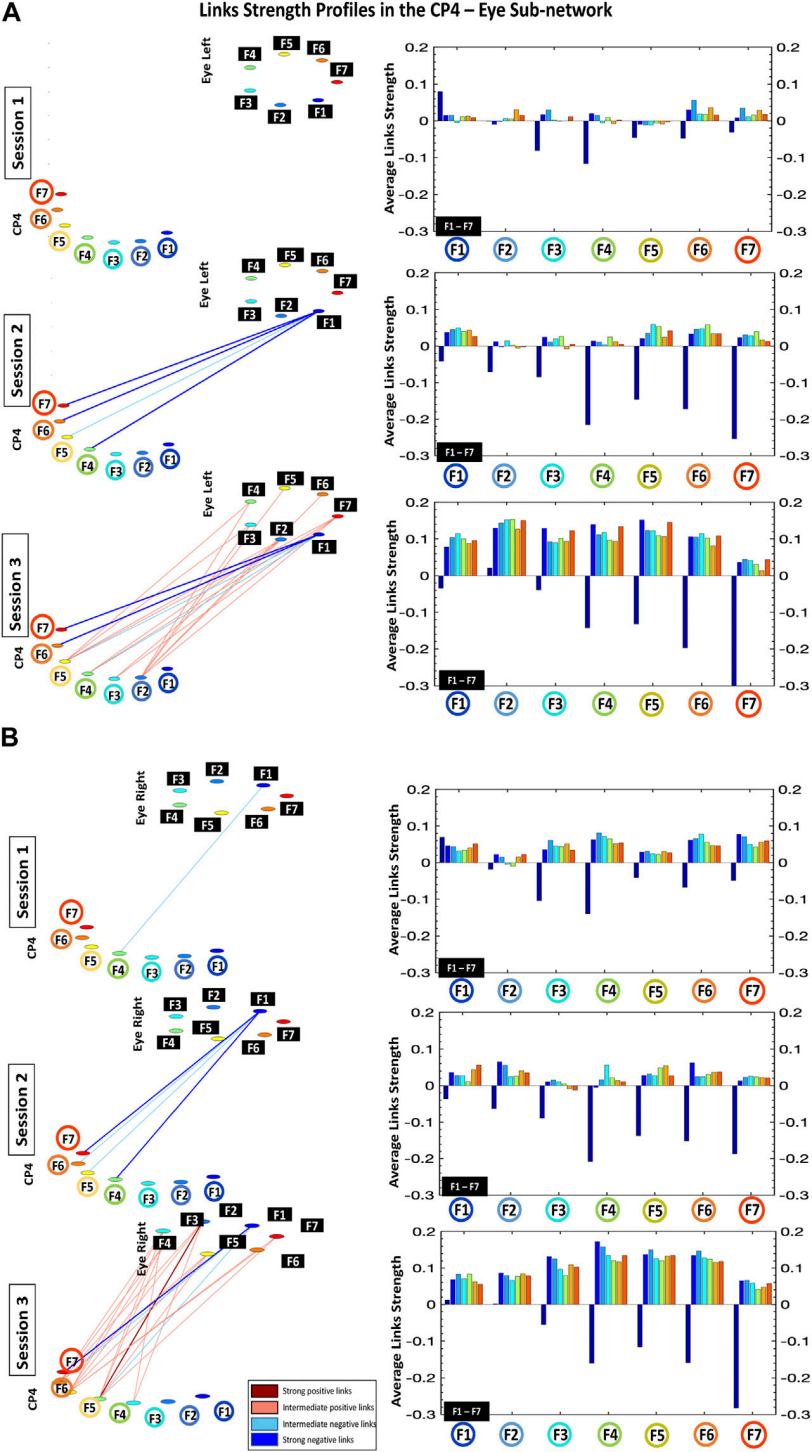
Links strength profiles in the CP4-Eye Left **(A)** and CP4-Eye Right **(B)** sub-networks during Session 1, Session 2, and Session 3. Left panels: networks represent the cross-correlation matrices (Figure 1), where network links correspond to the matrix elements and show the coupling strength (degree of synchronous activity) for the CP4-Eye sub-networks. Links strength is marked by line color and width (Section 2.5, *Methods*). Right panels: the CP4-Eye sub-networks topology is defined by basic modules, each representing the interaction of a given frequency band from CP4 with all frequency bands from Eye Left/Right. The frequency bands of CP4 are marked by circles on the horizontal axis of each bar chart, and the frequency bands of the Eye Left/Eye Right are marked by black squares within each module. Bars color in each profile corresponds to the color of the node associated with a given frequency band in the CP4 brain location.

To visualize the information provided by the cortico-ocular cross-correlation matrices, we mapped the matrices in Figure 1 into different networks for Session 1, Session 2, and Session 3. Each brain location and the Eye is represented by a semicircle/circle, where color nodes represent distinct frequency bands f_i_. Network links correspond to the values of cross-correlation matrix elements C_i,j_ in Figure 1. Link strength is marked by line color and width. To illustrate the differences in network organization, we used the following link strength classification: positive links [C_i,j_ > 0.17; dark red); weak positive links (0.12 < C_i,j_ < 0.17; light red); negative links (C_i,j_ < −0.17; dark blue)] and weak negative links (−0.17 < C_i,j_ < −0.12; light blue). Links corresponding to cross-correlation values −0.1< C_ij_ < 0.1 (Figure 2) are not shown in the network maps. Note that this set of thresholds was arbitrarily selected to provide the best visualization for the main results of the study shown in the cross-correlation matrices (Figure 1). To visualize the hierarchical organization of cortico-ocular network interactions among Brain Locations and Eyes, all multiplex networks (Figure 2) are presented separately as sub-network maps for all pairs of Brain Location-Eye (Figures 3, 4). Each sub-network map corresponds to a pair for a given Brain Location-Eye and follows the same color code as in the original network. Due to space limitations, we only present the sub-networks for the right hemisphere: P04-Eye Left, P04-Eye Right, CP4-Eye Left, and CP4-Eye Right (Figures 3, 4). The results for the left hemisphere (P03-Eye Left, and P03-Eye Right, CP3-Eye Left, CP3-Eye Right) are shown in Supplementary Figures S1, S2.

### 2.6 Statistical tests

To examine the changes in the relationships between the frequency bins of each subnetwork pair across time, we used a multi-step process. It should first be noted that the correlation between sub-network pairs at each time point was based on 900 observations (i.e., one observation every 2 seconds for each 30-min session), which rendered statistical significance even for very small correlations. Thus, we examined the changes in standardized estimates of association (i.e., Cohen’s *q*) across time. For all sub-networks examined, the correlation between frequency bin 1 for one node and frequency bin 7 for the second node of each pair changed considerably over time (see Supplementary Figure S3). For example, the correlation in the P04-Left Eye sub-network changed from 0.12 in Session 1 to −0.51 in Session 3.

## 3 Results

We identified and quantified a network of cortico-ocular interactions during NBA2K gaming and investigated changes in the synchronous activity of cortical/eye rhythms and network organization across repeated sessions. Below, we present the results per session.

### 3.1 Default network of cortico-ocular interactions (Session 1)

The default network of cortico-ocular interactions is characterized by a specific ensemble of interaction sub-networks (distinct heterogeneous matrices; Figure 1) representing all pairs of brain-eye interactions, where each sub-network exhibits a particular pattern of synchronization among cortical rhythms in the brain and frequency bands in the Eye Left/Eye Right. Specifically, all frequency bands for all brain locations and sub-networks are weakly and positively coupled with all frequency bands in the eyes (matrix elements in light blue and yellow colors; C ≈ 0.1; Figure 1), except for F1. In contrast, interactions between all frequency bands for all brain locations and F1 in the eyes (first column in the matrices; Figure 1) are markedly different: F3-F5 for all brain locations are negatively coupled with F1 in Eyes Left/Eyes Right (matrix elements in dark blue; C ≈ −0.1).

Accordingly, the network of cortico-ocular interactions (Figure 2) during Session 1 shows weak and very sparse links. Remarkably, the only visible links in the network are those representing negative interactions between F3-F5 in the different brain locations and F1 in both left and right eyes—low-frequency band F1 in the eyes represents the main mediator of interactions with the brain (see first dark blue bars for F3-F5; bar charts in Figures 3, 4).

### 3.2 Network of cortico-ocular interactions: Reorganization over time (Sessions 2 and 3)

Our analyses show that with repeated gaming sessions, the global cortico-ocular network (Figure 2) undergoes reorganization, where sub-networks representing pairs of Brain Location-Eye interactions (Figures 3, 4) exhibit a complex response to continuous play. Specifically, during Session 3 the network becomes i) denser with increased strength for positive links (≈300%; see matrix elements with warmer colors in Figure 1 and bar charts in Figures 3, 4) and ii) asymmetric—sub-networks in the right hemisphere (P04-Eye Left, P04-Eye Right, CP4-Eye Left, CP4-Eye Right) show stronger links compared to the left hemisphere (CP3-Eye Left, CP3-Eye Right, P03-Eye Left, P03-Eye Right; see Figures 1, 2).

Importantly, the negative links between F3-F5 in all brain locations and F1 in Eye Left/Eye Right observed in Session 1, are preserved for all sub-networks during Sessions 2 and 3 (Figures 3, 4). Further, additional negative and stronger links appear from Session 2 between F6-F7 for all brain locations and F1 in the left/right eyes—these links are approximately 300% more negative compared to Session 1 (Figures 3, 4). Note that during Session 3, low-frequency band F1 in the eyes still actuates as the main mediator of interactions with the brain (see first dark blue bars for F3-F7; bar charts in Figures 3, 4).

## 4 Discussion

Drawing from the field of Network Physiology, we used synchronized monitoring of two biological processes to explore an analytical approach that has not been applied previously for this specific problem: the interaction of the brain and the eyes in a game of a sports industry that is booming. In more detail, we presented preliminary results of the interactions of brain electrical and pupil diameter dynamics and brain in a male NBA2K gamer during several sessions. We demonstrated that each session is associated with a specific network of interactions (i.e., topology, node connectivity, number/strength of network links). Our results are the first empirical evidence on this kind of network and could become the basis of identifying universal behaviors (e.g., track how changes of one system can dynamically affect the behavior of other systems) in continuous play of sports simulation games.

Regarding the progress of our investigated system over time, our findings are specifically pointing toward one main direction: reorganization. On average, that reorganization was manifested *via* new interactions, F1 being a *hub*, hemispheric asymmetry (right over left), and a higher level of density (positive and negative links), reflecting increased network connectivity during Session 3.

Similar work has not been conducted in eSports. Therefore, we are not in a position to make direct comparisons with previous findings. However, our results are in accordance with findings from Network Physiology research. As previously described (Balagué et al., 2020), both underexpressed (weak) and overexpressed (strong) network connectivity could reflect dysfunctional/pathological states. More specifically, overexpressed/excessive connectivity as observed in Session 3, could be associated with a transitory underexpression of coupling network connectivity (i.e., imbalance: some processes are overexpressed and others underexpressed). Pathological conditions (e.g., neuro-muscular disorders) could increase the density and/or strength of interactions among certain nodes, pushing the system toward a rigid order which, in turn, could reduce its adaptability to environmental constraints (Ivanov et al., 2001; Stergiou et al., 2006; Stergiou and Decker, 2011). In addition, La Rocca et al. (2021) showed new brain-eye interactions based on stimuli (i.e., rest vs. task). In fact, the increase in functional connectivity (manifested intra- and inter-hemispherically) was strengthened with training. Similarly, Ivanov and Bartsch (2014) mapped a complex dynamic network including cerebral and ocular systems. As part of that work, transitions on a global and individual topological level were identified. To better understand the physiological mechanisms underlying cortico-ocular interactions in eSports, future studies should additionally assess how networks properties (degree of complexity, heterogeneity and asymmetry) change during the game. Techniques, such as Node-Based Multifractal Analysis (Xiao et al., 2021), would be suitable to encode the complex topology and structural interactions within a physiological networks.

Our brief research report is not immune to limitations. These are preliminary, observational, and exploratory data collected from one participant. Therefore, we cannot infer causality or generalize our findings. In addition, our EEG collected data from four channels only, limiting resolution, while the selected cortical locations (i.e., CP3, CP4, PO3, and PO4) are a delimitation related to equipment. Lastly, no data were collected regarding circadian rhythms, sleep, nutrition, or other potential confounders. Based on these shortcomings and the novelty of this research, we will avoid over-interpretation (e.g., provide specific physiological interpretations). For this reason, future studies should replicate this design using different video games and/or add more than one participant and/or days of playing. Alternative analysis methods (e.g., the Granger method, convex optimization, machine learning) are also suggested. Moreover, EEG with more channels/electrodes would increase the resolution and, thus, enhance the spatial sampling density. Additionally, the possibility of the observed network reorganization being related to player performance should be considered. Furthermore, causality cannot be inferred based on the design of our study. However, our study can stimulate future, hypothesis-driven studies to investigate the mechanisms (e.g., fatigue) of these dynamic relationships (e.g., regulation) and their effect on performance metrics (practice vs. competition). If, for example, fatigue is the main cause for this reorganization and that affects negatively the player’s rebounds per game, then these metrics could be used (in addition to existing markers; e.g., Djaoui et al., 2017) to proactively notify the NBA2K player/coach in real time (e.g., by capturing personal and environmental constraints) that they need a break/snack. Accumulation of fatigue is associated with *overtraining syndrome* (OTS). Although OTS is difficult to diagnose and treat effectively because it involves numerous factors that interact with each other in complex ways, Armstrong et al. (2022) recommend using a new approach to study OTS, known as complex systems analysis. That involves exploring patterns of interaction among various predisposing factors, rather than looking at individual factors in isolation. The two proposed methodological approaches to clarify the dynamics of physiological networks involved in OTS could potentially be used in future eSports research: Assessing cellular functions across multiple levels of complexity (e.g., the genome, epigenome, proteome, metabolome) and/or using machine learning to develop predictive models of OTS. In general, in a sport industry that grows at a pace we have never seen before, the practical implications of individualized, specific, adaptive, and periodized interventions (e.g., functional diversity) are numerous (Balagué et al., 2020). Additionally, further research is needed to explore cross-frequency coordination among other key organ systems (e.g., heart, muscle). Therefore, this framework can have not only practical but theoretical applications as it can lead toward the creation of general theories and principles about inter-organ interactions in eSports gaming. Lastly, *emergence* and *self-organization* are concepts that have garnered significant attention in statistical physics, and more recently, in the study of complex systems and complex networks, such as microbial communities and flocks of birds (e.g., Testa and Kier, 2000; De Wolf and Holvoet, 2005; Mariani et al., 2019). These phenomena are of great interest due to their ability to produce collective behavior and properties that cannot be explained solely by the interactions of individual components. The study of emergence and self-organization has thus become an important avenue for exploring the behavior and dynamics of complex systems, and has broad implications for various scientific fields. Can the phenomenon of reorganization be linked to the concepts of emergence and self-organization? While acknowledging the potential relevance of the concepts of self-organization and emergence to the current findings on physiological network dynamics, it is important to note that further investigation is needed to elucidate the nature and scope of these phenomena in the context of Network Physiology and eSports.

## Data Availability

The raw data supporting the conclusion of this article will be made available by the authors, without undue reservation.

## Supplementary material

SUPPLEMENTARY FIGURE S1
Links strength profiles in the CP3-Eye Left **(A)** and CP3-Eye Right **(B)** sub-networks during Session 1, Session 2, and Session 3. Left panels: networks represent the cross-correlation matrices (Figure 1), where network links correspond to the matrix elements and show the coupling strength (degree of synchronous activity) for the CP3-Eye sub-networks. Links strength is marked by line color and width (Sections 2, 2.5). Right panels: the CP3-Eye sub-networks topology is defined by basic modules, each representing the interaction of a given frequency band from CP3 with all frequency bands from Eye Left/Eye Right. Frequency bands of CP3 are marked by circles on the horizontal axis of each bar chart, and the frequency bands of the Eye Left/Eye Right are marked by black squares within each module. Bars color in each profile corresponds to the color of the node associated with a given frequency band in the CP3 brain location.

SUPPLEMENTARY FIGURE S2
Links strength profiles in the P03-Eye Left **(A)** and P03-Eye Right **(B)** during Session 1, Session 2, and Session 3. Left panels: dynamic cortico-ocular networks where network links show the coupling strength (degree of synchronous activity) for the P03-Eye sub-networks. Right panels: the P03-Eye sub-networks topology is defined by basic modules, each representing the interaction of a given frequency band from P03 with all frequency bands from Eye Left/Eye Right. Frequency bands of P03 are marked by circles on the horizontal axis of each bar chart, and the frequency bands of the Eye Left/Right are marked by black squares within each module. Bars color in each profile corresponds to the color of the node associated with a given frequency band in the P03 brain location.

SUPPLEMENTARY FIGURE S3

**(A,B)** Total Change in Standardized Correlation Estimates Across Time. In all correlograms, the y-axis represents the Eye (Left or Right) while the x-axis the Brain Location (CP3, P03, P04, and CP4). The darker the shade of red, the higher the total change. Note. The total change in standardized correlation estimates across time was calculated by taking the difference between the maximum and minimum observed correlations between frequency bands within each sub-network.
