# Supplementary figures and images for: Case report: Cortico-ocular interaction networks in NBA2K
Source: Front Netw Physiol. 2023 Apr 11;3:1151832. doi: 10.3389/fnetp.2023.1151832 (PMC10126506; doi:10.3389/fnetp.2023.1151832)

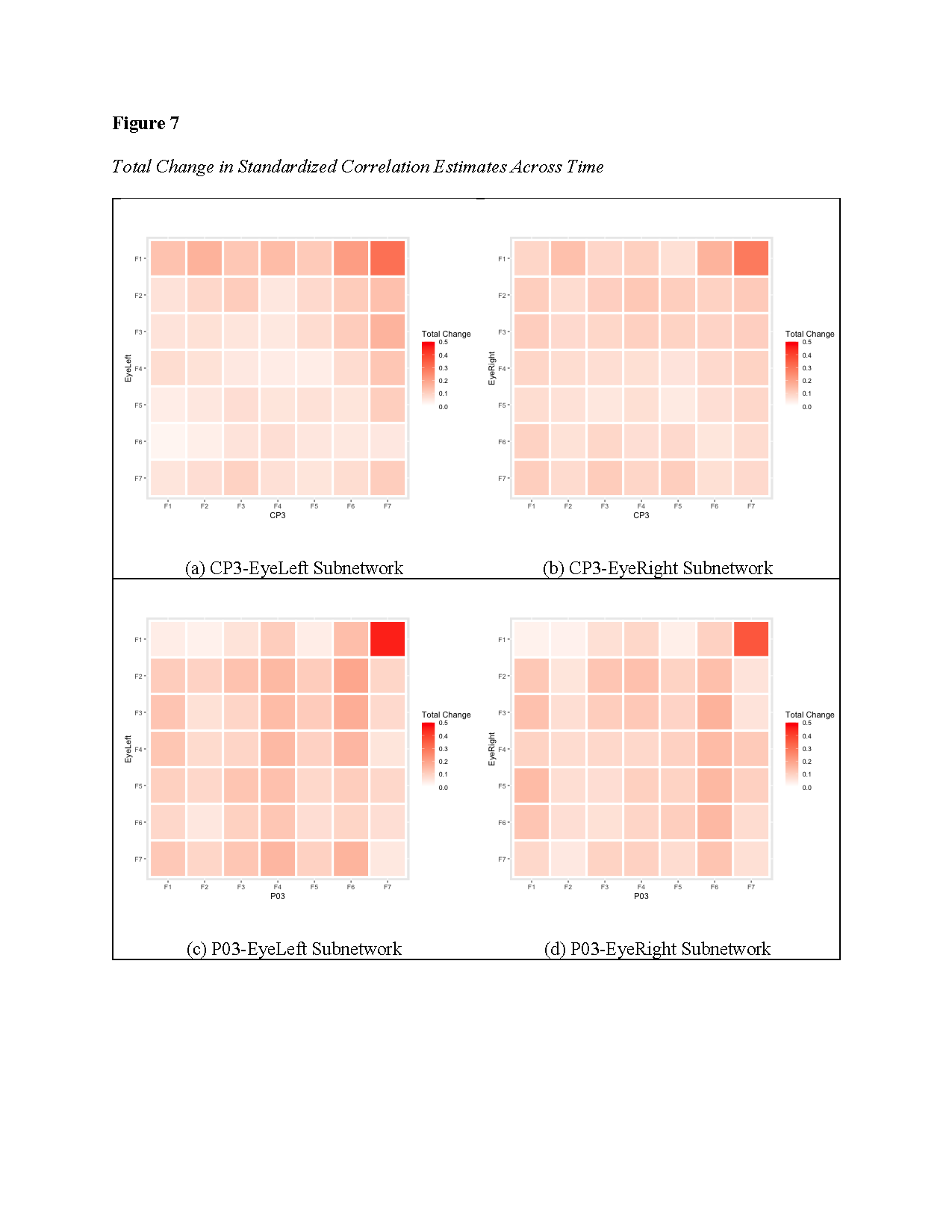

Supplement: Supplementary file 1 [file Image3.TIF]

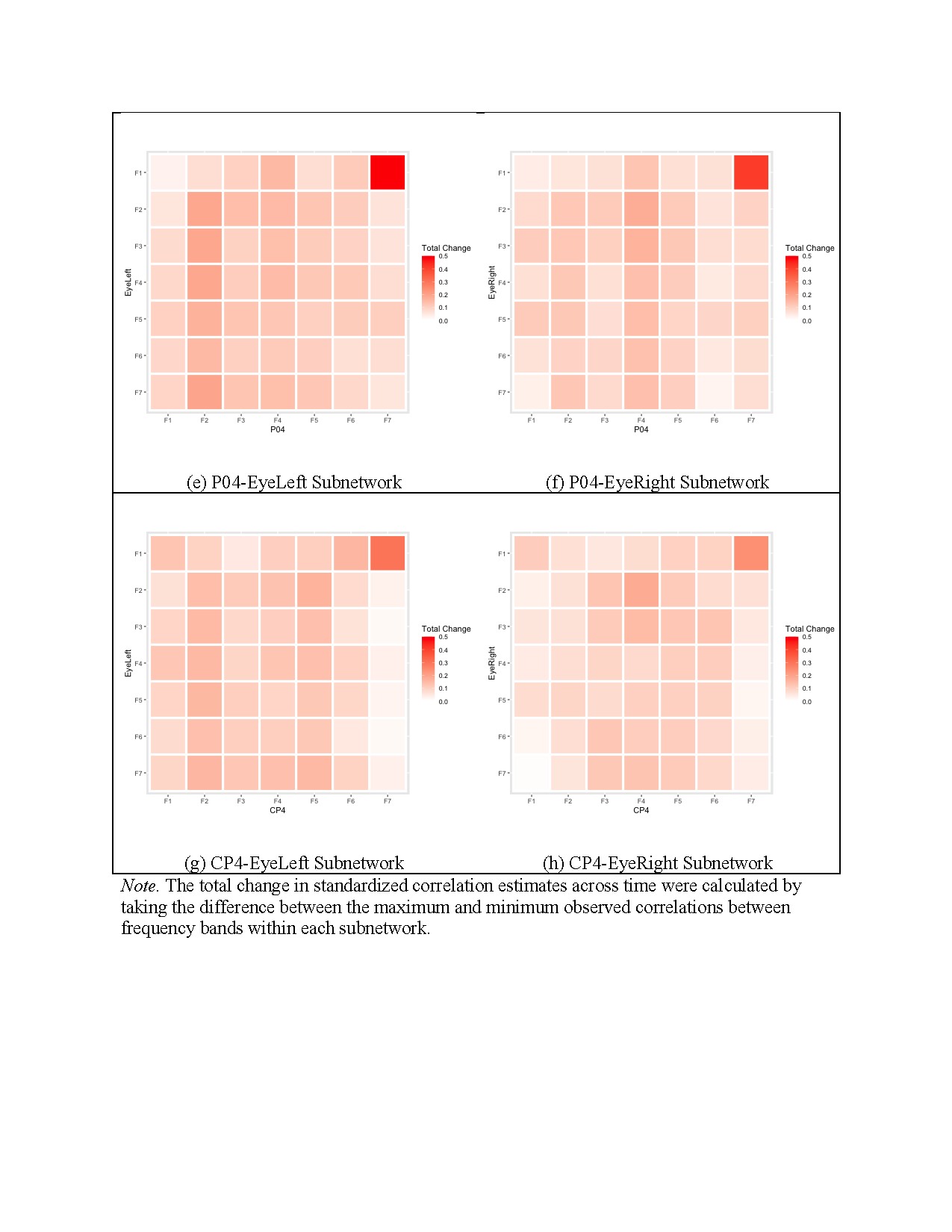

Supplement: Supplementary file 2 [file Image4.TIF]

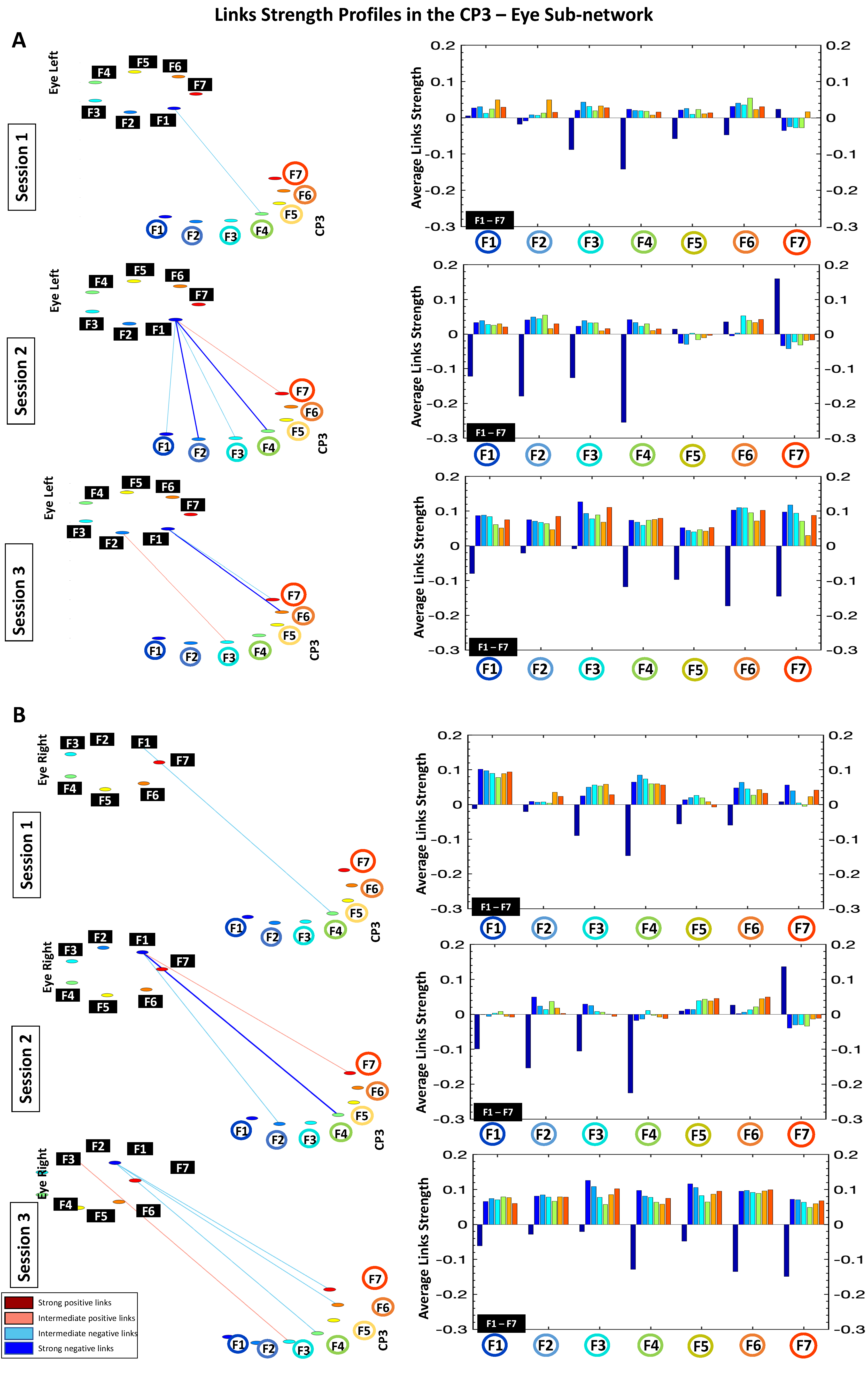

Supplement: Supplementary file 3 [file Image2.TIF]

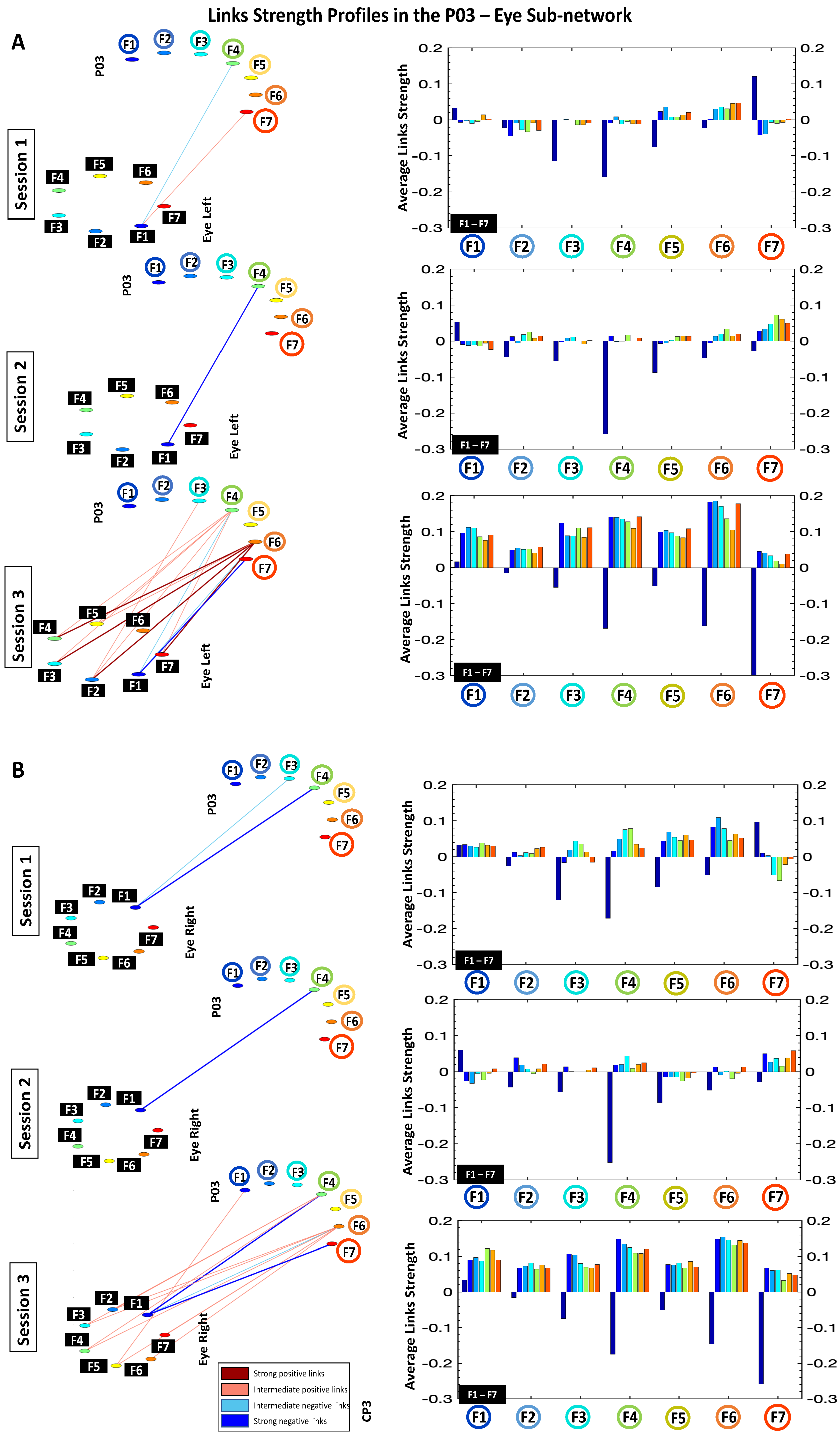

Supplement: Supplementary file 4 [file Image1.TIF]
