# Supplementary material for: Case report: Cortico-ocular interaction networks in NBA2K
Source: Front Netw Physiol. 2023 Apr 11;3:1151832. doi: 10.3389/fnetp.2023.1151832 (PMC10126506; doi:10.3389/fnetp.2023.1151832)
Supplement: Supplementary file 5 [file DataSheet1.docx]

The algorithm uses pandas EWM / exponentially weighted mean functionality located here:

https://pandas.pydata.org/docs/reference/api/pandas.DataFrame.ewm.html

Both pandas and TensorFlow use a smoothing factor that pandas calls 'alpha' we use a value of 0.3 in our implementation

This is equivalent to moving the smoothing slider in TensorFlow for those familiar with that.

Here is the relevant code section#====================================================================================================

# Smooth prior to downstream processing

#====================================================================================================

ts = 0.3 # tensorflow equivalent smoothing param

alpha = 1 - ts smoothed_left = df_gaze.LeftPupilFilled.ewm(alpha=alpha).mean()

smoothed_right = df_gaze.RightPupilFilled.ewm(alpha=alpha).mean() df_gaze['LeftPupilFinal'] = smoothed_left

df_gaze['RightPupilFinal'] = smoothed_right win_dur = 0.33

df_gaze['LeftPupilFinalRollingDiff'] = df_gaze.LeftPupilFinal.diff().rolling(int(FRAMERATE_GAZE*win_dur),center=True).mean()

df_gaze['RightPupilFinalRollingDiff'] = df_gaze.RightPupilFinal.diff().rolling(int(FRAMERATE_GAZE*win_dur),center=True).mean()
